# Primordial odontogenic tumor with prominent calcifications: A rare case report

**DOI:** 10.4317/jced.55925

**Published:** 2019-10-01

**Authors:** Sopee Poomsawat, Supak Ngamsom, Natee Nonpassopon

**Affiliations:** 1 DDS, MSc, PhD. Associate Professor, Department of Oral and Maxillofacial Pathology, Faculty of Dentistry, Mahidol University, Bangkok, Thailand; 2 DDS, MSc, PhD. Lecturer, Department of Oral and Maxillofacial Radiology, Faculty of Dentistry, Mahidol University, Bangkok, Thailand; 3 DDS. Lecturer, Mahachakri Sirindhorn Dental Hospital, Faculty of Dentistry, Mahidol University, Nakhon Pathom, Thailand

## Abstract

Primordial odontogenic tumor (POT) is a rare odontogenic tumor. It is a new entity in the latest edition of the World Health Organization classification in 2017. In the English-language literature, only 14 cases have been documented. Most POTs show a well-defined unilocular radiolucency surrounding a crown of an unerupted molar, resembling a dentigerous cyst. Microscopically, POT may be difficult to distinguish from odontogenic myxoma, ameloblastic fibroma, hyperplastic dental follicle and dental papilla. Here, we reported a case of POT in a 17-year old female presenting with an asymptomatic bony hard swelling at the left posterior mandible. Interestingly, this case shows unique radiographic and microscopic features with prominent calcifications and stellate reticulum-like structures. These characteristics have rarely been described in all previously reported POTs. Importantly, this case is the first case of POT demonstrating radiopacity in the radiographs. We encourage more cases of POTs to be documented as POTs may have more variations in radiographic and microscopic features. Importantly, oral radiologists, surgeons and pathologists must be aware of this new and rare tumor in order to avoid a misdiagnosis and an inappropriate treatment.

** Key words:**Calcification, mandible, odontogenic tumor, primordial odontogenic tumor.

## Introduction

Primordial odontogenic tumor (POT) is a rare odontogenic tumor first described in 2014 (1). It is a new entity in the latest edition of the World Health Organization (WHO) classification in 2017 (2). POT is classified as a benign mixed epithelial and mesenchymal odontogenic tumor. In the English-language literature, 14 cases have been documented (1,3-9). POT is a tumor of children and adolescents. The patient’s age ranges between 2 and 19 years, with the mean age of 11.2 years. A male predilection is noted (M: F= 1.8:1). The posterior mandible is the favorite site as 12 from 14 cases are found in this location. POT tends to present with an asymptomatic swelling. Radiographically, most cases show a well-defined unilocular radiolucency surrounding a crown of an unerupted tooth, resembling a dentigerous cyst. Microscopically, this tumor shows a well-circumscribed solid mass with a multilobulated pattern. The tumor consists of cells in a loose fibrous connective tissue, resembling the dental papilla that is covered by cuboidal or columnar cells resembling the inner enamel epithelium of the enamel organ (1,2). Calcification is very limited (3,6,7). To date, recurrences have never been recorded with the follow-up time ranging from 3 months to 20 years (4,6).

The aim of this study was to report an additional case of POT in order to document and better understand this rare tumor. Interestingly, this case shows unique radiographic and microscopic features with prominent calcifications and stellate reticulum-like structures. These characteristics have rarely been described in all previous cases. Importantly, this case is the first case of POT demonstrating radiopacity in the radiographs.

## Case Report

**Figure 1:**
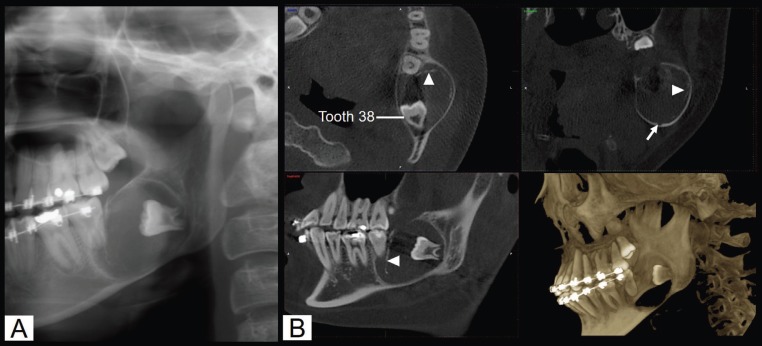
A) A cropped panoramic image shows a well-defined unilocular lesion with a corticated border, extending from the mesial root of the second molar to the ramus. A thin radiopaque band is noted at the anterior portion. B) Cone beam computed tomography (CBCT) images including axial, coronal, sagittal, and volume rendering reformation reveal a unilocular hypodensity lesion with buccal and lingual cortical bone expansions. A thinning of the inferior border of the mandible is observed. The inferior alveolar nerve canal (arrow) is displaced inferiorly. The radiopaque foci (arrowheads) are seen at the anterior portion.

A 17-year-old female with a left cheek swelling for 1 month was referred to our institute. Before this visit she was received two times incisional biopsy with inconclusive results. The patient did not experience pain or numbness of the lower lip. Her medical history was not relevant. Intra-oral examination revealed bony hard swelling at the buccal and lingual areas of the left mandible extending from distal area of the second mandibular molar to the ramus. The covering mucosa in this area was normal. The third mandibular molar was clinical crown absence. The second mandibular molar was a sound tooth with no mobility. Extra-oral examination showed only a slight facial asymmetry with a left facial enlargement. The cervical lymph nodes were within normal limit by manual examination. Radiographic findings demonstrated a well-defined, unilocular radiolucent lesion measuring 2.5x3.4 cm. associated with an unerupted third molar (Fig. 1). The internal structure showed radiopaque materials at the anterior portion. Bone expansions were noted. Root resorption was unremarkable.

A provisional diagnosis was a calcifying odontogenic cyst. Under local anesthesia, an incisional biopsy was performed at the distal area to the second mandibular molar. Microscopic examination of the specimen showed many spindle-shaped cells in a loose myxoid stroma. The histopathological diagnosis of odontogenic myxoma was made. Due to the histopathological result of odontogenic myxoma and the inadequate of the bone, partial mandibulectomy with iliac bone graft was performed. Then, the specimen was sent for microscopic examination. 

The received specimen was a part of the left mandible extending from the mesial aspect of the second mandibular molar to the ramus. A crown of the second mandibular molar was present in the specimen. Because the entire tumor was inside the mandible, the specimen was decalcified for a few days before cutting. The cut surface of a tumor showed a well- circumscribed multilobulated, whitish solid mass that was easily separated from the surrounding bone. No cystic cavities were revealed on serial sectioning. A crown of the third mandibular molar was embedded within the tumor mass.

**Figure 2:**
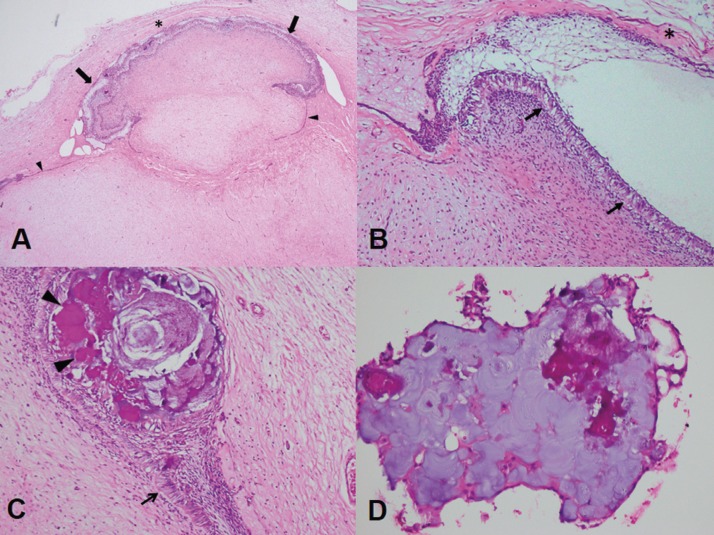
A) A multilobulated tumor mass consists of many spindle-shaped cells in a myxoid stroma, resembling dental papilla. This mass is frequently enveloped by epithelium resembling enamel organ (arrows) and is occasionally enveloped by a single layer of cuboidal epithelium (arrowheads). The fibrous capsule (*) of this tumor is clearly detected. There is no epithelial cells in the dental papilla-like myxoid tissue (H&E, x12.5) B) The epithelium, resembling the enamel organ, consists of the inner enamel epithelium-like columnar cells with reverse nuclear polarization (arrows) in association with several layers of stellate reticulum-like cells (H&E, x100). This epithelium is detected at the periphery of the tumor. The fibrous capsule (*) is present. C) This area of the tumor shows that many calcifications (arrowheads) are detected within the epithelium, resembling the enamel organ. An arrow indicates the inner enamel epithelium-like cells with reverse nuclear polarization (H&E, x100). D) A large sheet of calcified material shows concentric lamellar pattern. It is detached from the main tumor (H&E, x200).

Microscopic examination revealed an encapsulated, multilobulated tumor mass, composing of variable cellular myxoid connective tissue, resembling dental papilla. The periphery of the tumor was covered by a single layer of cuboidal epithelium or epithelium resembling an enamel organ of a tooth bud (Fig. 2A). The epithelium resembling the enamel organ consisted of inner enamel epithelium-like cells and stellate reticulum-like cells (Fig. 2B). Interestingly, many eosinophilic masses of varying degree of calcifications were found within the epithelium (Fig. 2C). Additionally, some calcified materials demonstrated concentric lamellar pattern. A large sheet of calcified material found in this case was somewhat similar to calcifications found in adenomatoid odontogenic tumors (Fig. 2D). The final diagnosis of POT was rendered. The operative was uneventful, and recurrence was not observed at 18 months follow up.

## Discussion

Based on the information in the English-language literature (1,3-9) and the latest WHO classification (2), the clinical, radiographic and microscopic features of our case have fulfilled the diagnosis of POT. The patient was a teenager presented with an asymptomatic bony hard swelling at the posterior mandible. Radiographs showed a unilocular radiolucency associated with an unerupted molar. Microscopic features showed dental papilla-like tissue enveloped by epithelium resembling inner enamel epithelium of the enamel organ. However, our case demonstrated prominent calcifications as perceived in the radiographs and in the microscopic slides. Additionally, this case showed remarkable stellate reticulum-like cells. Importantly, this is the first case of POT demonstrating radiopaque foci on the radiographs. All previous reports did not reveal radiopacity in the radiographs of POTs.

According to the English-language literature, 9 cases from 15 cases (including the present case) were reported from the Latin American region or were found in Hispanic patients (1,3,6). The remaining cases were from japan (2 cases) (5,7) and one case each from India (8), Egypt (4), the USA (9) and Thailand (the present case). The race of one case from the USA is not known (9). Therefore, it is possible that the pathogenesis of POT is influenced by genetic or environment. Geographic distribution of POTs may exist. Nevertheless, more cases of POTs are needed to draw this notion.

Radiographically, most POTs present with a unilocular radiolucency associated with an unerupted tooth. However, bilocular and multilocular appearances have also been reported. All lesions demonstrate bone expansion with a thining or perforation of cortical bone (1,3-9). Similar to most previous reports, the panoramic image and cone beam computed tomography (CBCT) images of our case showed an expansile, well-defined unilocular radiolucency associated with an unerupted molar. Nevertheless, this case revealed remarkable radiopaque foci within the lesion. This radiographic feature has never been previously demonstrated in all previous POTs. The presence of root resorption and displacement of surrounding structures on the radiographs of POTs are variable (1,3-9). In our case, the root resorption was unremarkable. However, the displacement of the inferior alveolar canal was depicted.

According to the presence of calcified materials on the radiographs, the differential diagnosis of our case focused on the group of odontogenic cysts or tumors with calcifications including calcifying odontogenic cyst or ameloblastic fibro-odontoma. This is in contrast to previous reports which give the differential diagnosis as dentigerous cyst, ameloblastoma, ameloblastic fibroma or unicystic ameloblastoma due to their radiolucent appearances (1,3,5,8,9). Therefore, we suggest that POT should be included in the differential diagnosis of a well-defined unilocular radiolucency with radiopaque foci particularly when it is associated with an unerupted molar. Additionally, POTs may show a variation in radiographic features.

Regarding the histopathological aspect, the stellate reticulum-like structures in our case were so common. We are aware that the stellate reticulum-like structure has not been described in the latest WHO classification (2). However, in this edition, it mentions that scant superficial layers of fusiform cells can be observed in some areas. Additionally, at least 5 cases of POT do contain stellate reticulum-like cells (3,5-7,9). The remarkable stellate reticulum-like cells in association with the inner epithelium-like cells of our case was similar to a POT recently reported by Bomfim *et al.* (6). The POT in Bomfim’s report (6) showed a well-circumscribed dental papilla-like mass covered by columnar cells resembling ameloblasts including the stellate reticulum-like cells. Therefore, our case and Bomfim’s case are different from the classic POT in that the inner enamel epithelium-like cells are associated with the stellate reticulum-like cells rather than present alone. The stellate reticulum-like cells in the other 4 cases of POT were not so prominent (3,5,7,9). Mikami *et al.* (7) found that the stellate reticulum-like cells were detected only in some areas. One case of POT showed that the surface epithelium consisting of inner enamel epithelium-like cells and only a few layer of polygonal cells (3). Based on the photographs in previous reports, two cases showed that the stellate reticulum-like cells were present as a thin layer and were not so prominent (5,9). Ando *et al.* (5) proposed that the stellate reticulum-like cells are fragile and thus they may be easily separated from the main tumor during the operation. Mikami and coworkers (7) hypothesized that the degree of differentiation within the epithelium in POTs may be different.

Besides the remarkable stellate reticulum-like structure, our case also contained many calcification masses. To date, only 3 cases of POTs contain calcifications as demonstrated in the microscopic slides. In contrast to our case, calcifications in those previous cases were very limited (3,6,7). In our case, calcifications were observed within the epithelial layer. A few large sheets of calcification were also noted. The amount and degree of calcifications found in the microscopic features were consistent with the findings in the radiographs. Since most calcifications were found within the epithelial layer, it is possible that the alkaline phosphatase within the epithelium contributes to these calcifications. In consistent with this notion, our case contained many epithelial cells and also a large amount of calcifications. The role of alkaline phosphatase in calcification process is evident (10). Additionally, it has been shown that epithelial tumor cells of calcifying epithelial odontogenic tumor exhibit alkaline phosphatase. This high alkaline phosphatase activity may be relevant to calcification production in this tumor (11-13). Further studies are needed to examine the presence of alkaline phosphatase in POTs.

The microscopic features of POT may be difficult to distinguish from odontogenic myxoma, ameloblastic fibroma, hyperplastic dental follicle and dental papilla (1,3,5,9). As occurred in the present case, the diagnosis of odontogenic myxoma was given from the incisional biopsy. The misdiagnosis of POT as odontogenic myxoma in our case affected options of procedures. After retrospectively reviewed the slides from the incisional biopsy, we did find a fragment of epithelium associated with a circular calcification mass besides the dental papilla-like tissue. Nevertheless, this fragment of epithelium was separated from the dental papilla-like mass. Thus, the relationship between the epithelium and the dental papilla-like tissue could not be evaluated. Furthermore, the epithelium was very tiny, and it was distorted resulting from the difficulty in cutting the specimen with a calcification mass. We agree with previous studies that the peripheral portion of POT is an important part that can be used to separate POT from odontogenic myxoma (1,3). The inner enamel epithelium-like tissue and the fibrous capsule are found only in POTs. However, this external portion of a tumor may not be included in the incisional biopsy. It is also possible that this thin layer of epithelium is destroyed during the operation. Therefore, to avoid the overtreatment, both oral surgeons and pathologists must be aware of POT when making a diagnosis of odontogenic myxoma. To differentiate these two lesions, a correlation with the clinical and radiographic features must be done meticulously. Although the favorite site of both lesions is the posterior mandible, odontogenic myxoma occurs in the third and the fourth decade of life (14). Odontogenic myxoma is rarely found associated with an unerupted tooth. Odontogenic myxoma does not have a fibrous capsule, and tumor cells may infiltrate the adjacent bone (2). Therefore, it is not easy to separate odontogenic myxoma from the surrounding bone. By contrast, POT shows a clear demarcation from the surrounding bone. These features may be helpful for oral surgeons.

The clinical features of POT and ameloblastic fibroma are quite similar. Thus, to separate these two lesions, the microscopic features play a key role. In ameloblastic fibroma, the mesenchymal element is more cellular, the cords of ameloblastic epithelium are distinct and distribute randomly within the mesenchymal element. Lastly, there is no epithelium surrounding the tumor mass (15). Unlike POT, dental papilla or hyperplastic dental follicle is not covered by ameloblastic epithelium. Additionally, the size of dental papilla or hyperplastic dental follicle is smaller than that of POT and may be a useful characteristic in making a differential diagnosis from POT (16,17).

## Conclusions

This study reported a case of POT, a rare odontogenic tumor. Our case is considered the fifteenth case of POTs in the English-language literature. Unlike most previous cases, this case showed prominent calcifications and stellate reticulum-like structures. Additionally, this is the first POT demonstrating radiopaque foci on the radiographs. We encourage more cases of POTs to be documented as POTs may have more variations in radiographic and microscopic features. Importantly, oral radiologists, surgeons, and pathologists must be aware of this new and rare tumor in order to avoid a misdiagnosis and an inappropriate treatment.
